# Vaccination discussions in community pharmacies following automated age-based screening of vaccination status through an appointment scheduling system

**DOI:** 10.1177/17151635251330844

**Published:** 2025-04-30

**Authors:** Sherilyn K.D. Houle, Saleema Bhaidani

**Affiliations:** School of Pharmacy, University of Waterloo, Waterloo, ON; MedEssist, Toronto, ON

## Introduction

Despite gains in population vaccination rates among Canadian adults over the past decade, many remain susceptible to vaccine-preventable disease. For example, vaccination rates from 2016 to 2023 for influenza among adults increased from 39.6% to 44.4%, pneumococcal disease among those age ≥65 years from 41.6% to 55%, herpes zoster (HZ) among those age ≥50 years from 20% to 38.8%, and human papillomavirus (HPV) from 25.2% to 46.9% among males age ≤26 years and 75.0% to 77.3% among females age ≤26 years.^1,2^ While the COVID-19 pandemic contributed to rising anti-vaccine sentiments often fueled by mis- and disinformation,^3^ health professional recommendations remain impactful, with 57.2% of unvaccinated adults reporting they would likely get vaccinated against HPV if recommended by their health provider, rising to over 70% for vaccines against pneumococcal disease and HZ.^2^

However, Canada’s currently strained primary care system can impact patient access to such recommendations, with 6.5 million Canadians lacking a family physician^4^ and pharmacists reporting that workload and time barriers negatively influenced their ability to proactively engage with patients on influenza vaccination.^5^

Previous research by our group reported on the use of automated prompts on users’ vaccination status built into appointment scheduling systems for influenza or COVID-19 vaccines in pharmacies. These prompts aimed to identify patients who were eligible for vaccination against HPV or HZ based on their age but who were unvaccinated, incompletely vaccinated, or uncertain of their vaccination status.^6^ Those who reported being not fully vaccinated or unsure were then prompted on whether they were interested in discussing their vaccination status with a pharmacist. If interest was expressed, a notation was added to the booking that was visible to pharmacy staff. Across 92,387 bookings from October 2021 to March 2022, 70.1% of users aged 9 to 45 years were potentially unvaccinated against HPV and 55.5% of those aged ≥50 years against HZ. Approximately 1 in 5 were interested in discussing these vaccines with a pharmacist.

The objective of the present study was to evaluate the outcomes of these automated additional vaccine prompts among patients who had scheduled a COVID-19 or influenza vaccination in community pharmacies.

## Methods

Data collection captured appointments made for individuals aged ≥9 years for influenza or COVID-19 vaccination at any community pharmacy across Canada using the MedEssist scheduling program between October 1, 2022 and March 5, 2024. Based on the age input while booking, users aged 9 to 45 years were prompted with optional questions on their vaccination status against HPV, users aged ≥50 years against HZ, and users aged ≥65 years against pneumococcal disease (an addition to the software after data collection for the previously published study), consistent with Canadian age-based recommendations for these vaccines.^7-9^ Those who reported being unvaccinated, incompletely vaccinated, or unsure of their vaccination status were further asked about their interest in speaking with a pharmacist about these vaccine(s). In addition to age, other data collected during the booking included the vaccine being scheduled (influenza or COVID-19) and self-reported gender.

If interest in discussing additional vaccination(s) with the pharmacist was selected during the booking, an icon indicating this was placed on the pharmacy-facing aspect of the MedEssist program. After each appointment, pharmacy staff users could confirm the appointment was complete in the system; however, this step was optional. When an appointment with expressed interest in having an additional discussion at the time of booking was confirmed as complete, pharmacy staff were asked, “Did you discuss other vaccines with the patient?” and if “Yes” was selected, the following questions were asked:

- How long was the discussion?
  - <2 minutes
  - 2 to 5 minutes
  - >5 minutes
- What was the outcome of the discussion?
  - Patient is interested in the vaccine, and steps have been made for the patient to receive the vaccine.
  - Patient is open to receiving the vaccine and plans to discuss it with their primary health care provider or do more research.
  - Patient is not interested in the vaccine.
  - Patient is not eligible or concerned about cost.
- Did the patient appear receptive to having a discussion about other vaccines with the pharmacy team?
  - Patient was receptive.
  - Patient was neutral.
  - Patient was unreceptive.

Microsoft Excel for Mac (version 16.86) was used for data analysis. User age was categorized as 9 to 17, 18 to 24, 25 to 29, 30 to 39, 40 to 45, 50 to 59, 60 to 69, or ≥70 years. Users aged 46 to 49 years were excluded from analysis as they did not meet age-based criteria for HPV, HZ, or pneumococcal vaccines. While the MedEssist program allowed users to select a preferred vaccine (e.g., high-dose influenza vaccine), vaccines were coded by indication regardless of the specific product documented. Entries with missing data related to patient age, vaccine booked, or vaccination conversation status were excluded, and any duplicate entries were identified and combined. Entries with missing data on vaccination history for HPV among the age 9 to 45 population were excluded, as well as entries missing data on vaccination history for both pneumococcal and HZ vaccines for the population age ≥50 years (an answer of “No” or “I don’t know” for at least 1 of these vaccines remained eligible for inclusion).

Patient demographics and conversation outcomes were calculated descriptively by age subgroup (9–45 years, ≥50 years, and ≥65 years), and the odds ratio (OR) and corresponding 95% confidence interval (CI) were calculated to examine associations between demographic characteristics and discussion outcomes. To facilitate analysis, we combined “patient is interested in the vaccine and steps have been made for the patient to receive the vaccine” and “patient is open to receiving the vaccine and plans to discuss it with their primary health care provider or do more research” into “interested or open to vaccination”, and we also combined “uninterested”, “undecided”, “ineligible”, or “concerned about cost” into “uninterested”.

A data-sharing agreement, including processes for ensuring anonymity of users and their responses to the screening questions, was established between the University of Waterloo and MedEssist. Ethics approval for the study was received from the University of Waterloo Office of Research Ethics (ORE 46433).

## Results

Over the study period, 140,566 vaccine appointments were scheduled across all age groups, with 91,066 including an age-based screening question. Of these, 9300 (10.2%) indicated willingness to have an additional discussion with a pharmacist, and 6674 appointments had the conversation status documented by pharmacy staff. Across 189 pharmacies, 1542 of these appointments were for patients aged 9 to 45 years, 5132 for those aged ≥50 years, and 2605 for those aged ≥65 years. Influenza vaccine comprised 3392 (50.8%) of appointments, while COVID-19 vaccine comprised 3282 (49.2%). Self-reported gender among our sample was missing for 17 patients, and 5 declined to answer, with the remainder distributed as follows: female (*n* = 3506, 52.5%), male (*n* = 3133, 46.9%), non-binary (*n* = 8, 0.1%), and other gender (*n* = 5, 0.07%).

Overall, 1523 of these conversations were marked as complete, representing 22.8% of all appointments. Of these, 221 (14.5%) documented information on the outcome of the encounter. Discussions were reported to take <2 minutes, 2 to 5 minutes, or >5 minutes in 116 (52.4%), 83 (37.6%), and 22 (10.0%) instances, respectively. Patients were perceived as receptive to recommendations in 196 (88.7%) encounters, neutral in 24 (10.9%), and unreceptive in 1 (0.4%). Following these discussions, 113 (51.1%) resulted in steps being made for the patient to receive the vaccine, 90 (40.7%) patients were open to vaccination and planned to discuss it with their primary care provider or do more research, 10 (4.5%) were not eligible for vaccination or were concerned about vaccine cost, and 8 (3.6%) were not interested in the vaccination(s) discussed. Descriptive results stratified by age subgroups (9–45 years or ≥50 years) are presented in Table 1. Appendix Table 1, found online in Supplementary Materials, presents the outcome of discussions (receptiveness and interest in vaccination) by age subgroup and the duration of the discussion, suggesting that short discussions (<2 minutes) are generally sufficient to encourage vaccination in this population.

**Table 1:** Patient demographics, discussions held, and outcomes by age subgroups

Variable	Age 9–45 years (*n* = 1542)	Age ≥50 years (*n* = 5132)	Age ≥65 years (*n* = 2605)
**Age in years (mean, SD)**	31.3 (10.5)	65.4 (9.1)	72.6 (6.4)
**Gender, *n* (%)**			
Female	782 (50.7)	2724 (53.1)	1317 (50.6)
Male	742 (48.1)	2391 (46.6)	1278 (49.1)
Non-binary	7 (0.4)	1 (0.02)	0
Prefer not to answer	4 (0.3)	1 (0.02)	0
Other gender	4 (0.3)	2 (0.03)	1 (0.04)
Missing	3 (0.2)	13 (0.3)	9 (0.4)
**Vaccine booked, *n* (%)**			
Influenza	815 (52.9)	2577 (50.2)	1390 (53.4)
COVID-19	727 (47.1)	2555 (49.8)	1215 (46.6)
**HPV vaccination status, *n* (%)**			
Not vaccinated	930 (60.3)	Not applicable	
Unsure	612 (39.7)		
**HZ vaccination status, *n* (%)**			
Not vaccinated	Not applicable	3428 (66.8)	1531 (58.7)
Unsure		328 (6.4)	221 (8.5)
Yes		1374 (26.8)	853 (32.7)
Missing		2 (0.04)	0
**Pneumococcal vaccination status, *n* (%)**			
Not vaccinated	Not applicable	Not applicable	1612 (61.9)
Unsure			629 (24.1)
Yes			353 (13.6)
Missing			11 (0.4)
**Discussion held on additional vaccination(s), *n* (%)**			
Yes	312 (20.2)	1209 (23.6)	638 (24.5)
No	1230 (79.8)	3923 (76.4)	1967 (75.6)
**Length of discussion, *n* (%) of discussions with outcome data reported**			
<2 minutes	19 (42.2)	97 (55.1)	50 (50.1)
2–5 minutes	23 (51.1)	60 (34.1)	15 (15.2)
>5 minutes	3 (66.7)	19 (10.8)	34 (34.3)
**Receptiveness of patient to vaccination, *n* (%) of discussions with outcome data reported**			
Receptive	39 (86.7)	157 (89.2)	92 (93.0)
Neutral	6 (13.3)	18 (10.2)	7 (7.0)
Not receptive	0	1 (0.6)	0
**Outcome of discussion, *n* (%) of discussions with outcome data reported**			
Action taken toward vaccination	14 (31.1)	99 (56.3)	59 (60.0)
Interested, but wishes to consult other health professional or do research	27 (60.0)	63 (35.8)	35 (35.4)
Not eligible or concerned about cost	2 (4.4)	8 (4.5)	2 (2.0)
Not interested	2 (4.4)	6 (3.4)	3 (3.0)

HPV, human papillomavirus; HZ, herpes zoster; SD, standard deviation.

Statistically significant predictors of having a discussion on additional vaccines included a negative relationship with scheduling an appointment for COVID-19 vaccination vs influenza among both the ≥50 years (OR, 0.82; 95% CI, 0.72–0.94) and the ≥65 years (OR, 0.76; 95% CI, 0.63–0.91) subgroups as well as a positive relationship with those declining to specify their gender orientation vs male among both the ≥50 years (OR, 21.20; 95% CI, 4.77–94.24) and the ≥65 years (OR, 24.2; 95% CI, 3.02–194.7) subgroups (Table 2). Age 50 to 59 years was associated with lower odds of being receptive to the discussion than age ≥70 years (OR, 0.16; 95% CI, 0.03–0.81).

**Table 2:** Odds ratio estimates of having a discussion on additional vaccines by patient characteristics among those who answered “no” or “uncertain” to additional vaccination screening question(s) and “yes” to interest in discussing vaccination with the pharmacist

Characteristic	Age 9–45 years, OR (95% CI)	Age ≥50 years, OR (95% CI)	Age ≥65 years, OR (95% CI)
**Discussion held on additional vaccines (yes vs no)**			
Booking for COVID-19 vaccine vs influenza	0.80 (0.62–1.02)	0.82 (0.72–0.94)*	0.76 (0.63–0.91)*
Age (years)			
9–17 vs 40–45	0.79 (0.53–1.19)	Not applicable	
18–24 vs 40–45	0.79 (0.47–1.33)		
25–29 vs 40–45	1.03 (0.65–1.65)		
30–39 vs 40–45	0.97 (0.71–1.32)		
50–59 vs ≥70	Not applicable	0.88 (0.74–1.04)	Not applicable
60–69 vs ≥70		1.06 (0.91–1.23)	1.10 (0.92–1.32)
Gender			
Female vs male	1.19 (0.93–1.53)	1.13 (0.87–1.13)	0.94 (0.79–1.13)
Non-binary or other orientation vs male	0.43 (0.05–3.39)	0.002 (0.20–52.24)	Unable to calculate^†^
Prefer not to say vs male	0.72 (0.09–6.00)	21.20 (4.77–94.24)*	24.2 (3.02–194.7)*
HPV vaccination status (unsure vs not vaccinated)	1.22 (0.95–1.57)	Not applicable	
HZ vaccination status			
Unsure vs not vaccinated	Not applicable	1.18 (0.91–1.57)	1.25 (0.91–1.71)
Yes vs not vaccinated		1.10 (0.95–1.28)	0.99 (0.82–1.21)
Pneumococcal vaccination status			
Unsure vs not vaccinated	Not applicable	0.89 (0.72–1.11)	0.89 (0.72–1.11)
Yes vs not vaccinated		1.07 (0.82–1.39)	1.07 (0.82–1.39)
**Patient receptive to recommendation (receptive vs neutral or not receptive)**			
Booking for COVID-19 vaccine vs influenza	0.89 (0.14–5.53)	0.77 (0.27–2.15)	0.44 (0.09–2.14)
Age (years)			
9–17 vs 40–45	0.91 (0.07–12.52)	Not applicable	
18–24 vs 40–45	Unable to calculate^†^		
25–29 vs 40–45	Unable to calculate^†^		
30–39 vs 40–45	1.03 (0.15–7.19)		
50–59 vs ≥70	Not applicable	0.16 (0.03–0.81)*	Not applicable
60–69 vs ≥70		0.48 (0.09–2.46)	2.00 (0.37–10.90)
Gender			
Female vs male	1.90 (0.31–11.6)	0.52 (0.18–1.52)	0.96 (0.20–4.52)
Non-binary or other orientation vs male	Unable to calculate^†^	Unable to calculate^†^	Unable to calculate^†^
Prefer not to say vs male	Unable to calculate^†^	Unable to calculate^†^	Unable to calculate^†^
HPV vaccination status (unsure vs not vaccinated)	3.2 (0.52–19.67)	Not applicable	
HZ vaccination status			
Unsure vs not vaccinated	Not applicable	1.18 (0.91–1.57)	Unable to calculate^†^
Yes vs not vaccinated		1.10 (0.95–1.28)	Unable to calculate^†^
Pneumococcal vaccination status			
Unsure vs not vaccinated	Not applicable	0.89 (0.72–1.11)	1.23 (0.13–11.6)
Yes vs not vaccinated		1.07 (0.82–1.39)	0.29 (0.05–1.82)
**Patient interest in vaccination (interested or open vs undecided, uninterested, or ineligible)**			
Booking for COVID-19 vaccine vs influenza	Unable to calculate^†^	1.37 (0.36–5.13)	1.45 (0.15–13.60)
Age (years)			
9–17 vs 40–45	Unable to calculate^†^	Not applicable	
18–24 vs 40–45	Unable to calculate^†^		
25–29 vs 40–45	Unable to calculate^†^		
30–39 vs 40–45	Unable to calculate^†^		
50–59 vs ≥70	Not applicable	0.57 (0.16–2.02)	Not applicable
60–69 vs ≥70		2.48 (0.57–10.85)	Unable to calculate^†^
Gender			
Female vs male	2.86 (0.27–29.8)	0.60 (0.18–2.00)	0.85 (0.13–5.30)
Non-binary or other orientation vs male	Unable to calculate^†^	Unable to calculate^†^	Unable to calculate^†^
Prefer not to say vs male	Unable to calculate^†^	Unable to calculate^†^	Unable to calculate^†^
HPV vaccination status (unsure vs not vaccinated)	1.41 (0.18–11.03)	Not applicable	
HZ vaccination status			
Unsure vs not vaccinated	Not applicable	0.52 (0.10–2.68)	Unable to calculate^†^
Yes vs not vaccinated		0.99 (0.25–3.86)	0.58 (0.09–3.67)
Pneumococcal vaccination status			
Unsure vs not vaccinated	Not applicable	0.59 (0.05–6.91)	0.59 (0.05–6.91)
Yes vs not vaccinated		0.14 (0.02–1.13)	0.14 (0.02–1.13)

* *p* < 0.05. ^†^An odds ratio could not be calculated as one of the odds’ denominators was zero.

CI, confidence interval; HPV, human papillomavirus; HZ, herpes zoster; OR, odds ratio.

Of all discussions with documented outcomes, only 1 patient appeared to refuse vaccination in general: a female aged 60 to 69 who was unvaccinated against both HZ and pneumococcal disease. This conversation was reported to have taken less than 2 minutes, with the patient being unreceptive and uninterested in vaccination.

## Discussion

Automated age-based prompts built into vaccination scheduling software can identify opportunities for discussions between patients and pharmacists on vaccinations other than those being scheduled. These discussions are often brief and, in over 90% of documented encounters, result in a decision to be vaccinated or openness to vaccination following patient-pursued additional research or consultation with another primary care provider.

Canadian pharmacists feel they play an important role in vaccination efforts and are confident in their ability to identify individuals with vaccine hesitancy, engage in conversation, and respond to their concerns but are limited in their ability to engage in these discussions by workload, time, and staffing.^5^ However, pharmacists have also been found to conflate vaccine hesitancy with a binary position (anti-vaccine vs pro-vaccine), which may contribute to reluctance to proactively bring up vaccination with patients out of fear of encountering resistance.^10^ A recent survey of Canadian parents reports findings consistent with other research that has positioned vaccine hesitancy as a continuum ranging from complete acceptance to complete refusal, with most individuals residing in the middle.^11^ Specifically, this survey categorized 29% of respondents at the acceptance end of the continuum, 14% at the refusal end, and the remainder being largely supportive with some reservation or skeptical but not dismissive.^12^ Pharmacists should anticipate willingness to discuss vaccination—as most patients will be receptive—but should also efficiently identify those with a currently unmovable position and respectfully end the conversation. Acknowledging the patient’s decision and indicating willingness to discuss vaccination in the future should they reconsider will maintain the patient–pharmacist relationship, avoid engaging in argument, and allow for scarce time to be allocated to those who are receptive.

Limitations of this work include the likelihood of bias towards patients with a positive view of vaccination as they have both scheduled a vaccination appointment and indicated willingness to engage in additional discussion. Data are also unavailable on the impact of the prompts on the 77.2% of appointments where interest in a discussion was noted but the appointment was not confirmed to be complete by pharmacy staff, including whether a discussion was offered and, if so, reasons provided by patients for declining or the outcomes of accepted but undocumented discussions. Screening questions were strictly age-based and did not consider clinical criteria that may impact vaccine indications. Odds ratio calculations were restricted by small sample sizes within subgroups, which precluded the calculation of odds ratios for outcomes with no events in a denominator or resulted in wide confidence intervals. Finally, the impact of similar prompts within appointments booked for the administration of other vaccines, such as travel vaccines, is also uncertain, as evidence has consistently shown high acceptance rates for recommended travel vaccines.^13-15^ Coupling routine vaccination assessment with pre-travel consultations is recommended^16^ and may further support efforts to optimize overall vaccine uptake.

Despite the identification of 9300 potential vaccine discussion candidates, only a small proportion of all bookings resulted in a documented conversation (i.e., 1523/9300, only 16%). This may be due to a number of factors, including public hesitancy to provide optional information online, low visibility of the notification on the pharmacy-facing interface, or pharmacist hesitancy to initiate a conversation due to concerns of encountering vaccine hesitancy or of additional workload. Program modifications such as providing users with access to information on the vaccines mentioned in the software to review before expressing interest in a conversation, posing screening questions again in appointment confirmation, reminder emails for those who did not complete the questions during booking, and higher visibility notifications for pharmacy staff are strategies to consider for product updates.

## Conclusion

Vaccine discussions coupled to existing vaccination appointments are usually brief but effective, demonstrating that even short interactions can positively influence vaccination decisions and that most patients screened in this manner are receptive to a pharmacist’s recommendation and prepared to take action to receive a recommended vaccine. Automated integration of vaccination screening questions into appointment booking software for other pharmacy services can raise awareness of other indicated vaccines a patient may be eligible for while also being more integrated into the existing workflow than manual screening, supporting pharmacists in offering this important public health service.

## Supplemental Material

sj-pdf-1-cph-10.1177_17151635251330844 – Supplemental material for Vaccination discussions in community pharmacies following automated age-based screening of vaccination status through an appointment scheduling system Supplemental material, sj-pdf-1-cph-10.1177_17151635251330844 for Vaccination discussions in community pharmacies following automated age-based screening of vaccination status through an appointment scheduling system by Sherilyn K.D. Houle and Saleema Bhaidani in Canadian Pharmacists Journal / Revue des Pharmaciens du Canada

## References

[1] Public Health Agency of Canada. Vaccine uptake in Canadian adults: Results from the 2016 Adult National Immunization Coverage Survey (aNICS); July 2018. Available: https://publications.gc.ca/collections/collection_2018/aspc-phac/HP40-222-2018-eng.pdf (accessed Feb. 28, 2025).

[2] Public Health Agency of Canada. Adult National Immunization Coverage Survey (aNICS): 2023 results; January 2024. Available: https://www.canada.ca/en/public-health/services/immunization-vaccines/vaccination-coverage/adult-national-immunization-coverage-survey-2023-results.html (accessed Feb. 28, 2025).

[3] To QG To KG Huynh V-AN , et al. Anti-vaccination attitude trends during the COVID-19 pandemic: A machine learning-based analysis of tweets. Digit Health 2023;9:1-9. doi:10.1177/20552076231158033. PMC9941594 36825077

[4] Duong D Vogel L . National survey highlights worsening primary care access. Can Med Assoc J 2023;195(16):E592-E593. doi:10.1503/cmaj.1096049. PMC10125184 37094873

[5] Pullagura GR Violette R Houle SKD Waite NM . Exploring influenza vaccine hesitancy in community pharmacies: Knowledge, attitudes, and practices of community pharmacists in Ontario, Canada. Can Pharm J 2020;153(6):361-70. doi:10.1177/1715163520960744. PMC7689625 33282027

[6] Houle SKD Alsabbagh MW Waite NM . Herpes zoster and human papillomavirus vaccination opportunities identified using electronic prompts at the time of scheduling influenza or COVID-19 vaccines. Can Pharm J 2023;156(5):257-64. doi:10.1177/17151635231188343. PMC10786011 38222890

[7] Public Health Agency of Canada. Human papillomavirus (HPV) vaccines: Canadian Immunization Guide; July 2024. Available: https://www.canada.ca/en/public-health/services/publications/healthy-living/canadian-immunization-guide-part-4-active-vaccines/page-9-human-papillomavirus-vaccine.html (accessed Feb. 28, 2025).

[8] Public Health Agency of Canada. Herpes zoster (shingles) vaccine: Canadian Immunization Guide; October 2023. Available: https://www.canada.ca/en/public-health/services/publications/healthy-living/canadian-immunization-guide-part-4-active-vaccines/page-8-herpes-zoster-(shingles)-vaccine.html (accessed Feb. 28, 2025).

[9] Public Health Agency of Canada. Pneumococcal vaccines: Canadian Immunization Guide; May 2024. Available: https://www.canada.ca/en/public-health/services/publications/healthy-living/canadian-immunization-guide-part-4-active-vaccines/page-16-pneumococcal-vaccine.html (accessed Feb. 28, 2025).

[10] Pullagura GR Violette R Houle SKD Waite NM . Shades of gray in vaccination decisions—Understanding community pharmacists’ perspectives of, and experiences with, influenza vaccine hesitancy in Ontario, Canada. Vaccine 2020;38(11):2551-58. doi:10.1016/j.vaccine.2020.01.085. 32037223

[11] Larson HJ Jarrett C Eckersberger E Smith DMD Paterson P . Understanding vaccine hesitancy around vaccines and vaccination from a global perspective: A systematic review of published literature, 2007-2012. Vaccine 2014;32(19):2150-59. 10.1016/j.vaccine.2014.01.081 24598724

[12] Angus Reid Institute. Parental opposition to childhood vaccination grows as Canadians worry about harms of anti-vax movement; February 2024. Available: https://angusreid.org/canada-vaccines-childhood-vaccinations-anti-vax-mandates-covid-19-flu-mmr-side-effects/ (accessed Feb. 28, 2025).

[13] Hess KM Dai C Garner B Law AV . Measuring outcomes of a pharmacist-run travel health clinic located in an independent community pharmacy. J Am Pharm Assoc 2010;50:174-80. 10.1331/JAPhA.2010.09204 20199959

[14] Tran D Gatewood S Moczygemba LR , et al. Evaluating health outcomes following a pharmacist-provided comprehensive pretravel health clinic in a supermarket pharmacy. J Am Pharm Assoc 2015;55:143-52. 10.1331/JAPhA.2015.14140 25749263

[15] Houle SKD Bascom CS Rosenthal MM . Clinical outcomes and satisfaction with a pharmacist-managed travel clinic in Alberta, Canada. Trav Med Infect Dis 2018;23:21-26. 10.1016/j.tmaid.2018.02.011 29486241

[16] Chen LH Hochberg N . CDC Yellow Book 2024: The pretravel consultation; May 2023. Available: https://wwwnc.cdc.gov/travel/yellowbook/2024/preparing/pretravel-consultation (accessed Feb. 28, 2025).

