# Supplementary material for: Vaccination discussions in community pharmacies following automated age-based screening of vaccination status through an appointment scheduling system
Source: Can Pharm J (Ott). 2025 Apr 30;158(4):206–12. doi: 10.1177/17151635251330844 (PMC12043598; doi:10.1177/17151635251330844)
Supplement: sj-pdf-1-cph-10.1177_17151635251330844 – Supplemental material for Vaccination discussions in community pharmacies following automated age-based screening of vaccination status through an appointment scheduling system [file sj-pdf-1-cph-10.1177_17151635251330844.pdf]

APPENDIX: TABLE 1 Outcome of vaccine discussion by duration of discussion and age subgroup

Duration of discussion	Patient receptive (n, %)	Interest in vaccination (n, %)
**Age 9–45 years**		
<2 minutes (n = 19)	16 (84.2%)	17 (89.5%)
2–5 minutes (n = 23)	21 (91.3%)	22 (95.7%)
>5 minutes (n = 3)	2 (66.7%)	2 (66.7%)
**Age ≥50 years**		
<2 minutes (n = 97)	85 (87.6%)	85 (87.6%)
2–5 minutes (n = 60)	54 (90.0%)	59 (98.3%)
>5 minutes (n = 19)	18 (94.7%)	18 (94.7%)
**Age ≥65 years**		
<2 minutes (n = 50)	47 (94.0%)	47 (94.0%)
2–5 minutes (n = 34)	31 (91.2%)	33 (97.1%)
>5 minutes (n = 15)	14 (93.3%)	14 (93.3%)

Houle SKD, Shaidani S. Vaccination discussions in community pharmacies following automated age-based screening of vaccination status through an appointment scheduling system. *Can Pharm J (Ott)* 2025;158. DOI 10.1177/17151635231330844.
